# Human disturbance impacts the integrity of sacred church forests, Ethiopia

**DOI:** 10.1371/journal.pone.0212430

**Published:** 2019-03-06

**Authors:** Catherine L. Cardelús, Carrie L. Woods, Amare Bitew Mekonnen, Sonya Dexter, Peter Scull, Berhanu Abraha Tsegay

**Affiliations:** 1 Department of Biology, Colgate University, Hamilton, NY, United States of America; 2 Department of Biology, University of Puget Sound, Puget Sound, WA, United States of America; 3 Department of Biology, Bahir Dar University, Bahir Dar, Ethiopia; 4 Department of Geography, Colgate University, Hamilton, NY, United States of America; Pacific Northwest National Laboratory, UNITED STATES

## Abstract

Land-use change can have profound effects on forest communities, compromising seedling recruitment and growth, and long-term persistence of forests on the landscape. Continued forest conversion to agriculture causes forest fragmentation which decreases forest size, increases edge effects and forest isolation, all of which negatively impact forest health. These fragmentation effects are magnified by human use of forests, which can compromise the continued persistence of species in these forests and the ability of the forests to support the communities that depend on them. We examined the extent and influence of human disturbance (e.g. weedy taxa, native and exotic tree plantations, clearings, buildings) on the ecological status of sacred church forests in the northern highlands of South Gondar, Ethiopia and hypothesized that disturbance would have a negative effect. We found that disturbance was high across all forests (56%) and was negatively associated with tree species richness, density, and biomass and seedling richness and density. Contrary to expectation, we found that forests < 15.5 ha show no difference in disturbance level with distance from population center. Based on our findings, we recommend that local conservation strategies not only protect large forests, but also the small and highly used forests in South Gondar which are critical to the needs of local people, including preserving large trees for seed sources, removing exotic and weedy species from forests, and reducing clearings and trails within forests.

## Introduction

Isolation and degradation can be the silent demise of forests as towering canopy trees give the impression of a healthy forest ecosystem, while understory species composition can be dominated solely by pioneer species, completely void of seedlings from old growth species. This phenomenon has been written about in numerous ways, most famously by Janzen [1] who characterized latent extinction as single, majestic, long-lived trees stranded in vast agrospace or a forest of trees that can no longer reproduce, but survive as living dead. Deforestation and degradation, still rampant in tropical regions [2], are not the only form of forest loss. The relentless destruction of buffer zones around forests increases forest isolation and edge effects, such as increased wind and decreased humidity [3–5], and can be devastating to overall forest integrity and ecology as they can magnify the disturbance within the forest. Disturbances within forests, for example grazing animals that trample seedlings and compact the soil, can also lead to forest decline, through forces such as reduced species richness and density, both of which compromise regeneration potential [4, 6–10]. A recent metanalysis on forest isolation and edge effects indicates that 70% of the world’s remaining forests are within 1000 m of the edge, with 20% of those within 100 m of an edge [11].

Land use change (LUC) continues in tropical countries, with a conservative estimate from the Food and Agricultural Organization of 8 million ha deforested annually from 2000–2010 [2]. The human influence on this is clear as 6 million ha of these forests were converted to agricultural land [2]. Smaller scale human impacts on forests such as timber and non-timber product harvesting [12], planting of exotic species (e.g. *Eucalyptus;* [13]), and live-stock grazing, and clearings or gathering areas [8] can have pervasive impacts on forests. The myriad impacts of LUC on the local, regional and global scales are well documented such as the loss of biodiversity [14, 15], decreased carbon storage, varying weather patterns [16], increased fire frequency and intensity [17], and loss of other ecosystem services [15, 18]. Land use change also impacts the communities that use forest resources; as resources diminish, cultural traditions can be compromised or lost [19]. These effects can be felt more acutely in regions that rely on the land and are socioeconomically disadvantaged.

Forest size, the degree of disturbance, the presence of roads and distance to population center can also contribute to forest degradation. In tropical regions, roads are cited as the “first of a hundred cuts” that degrade intact forests, as they allow the initial penetration of the forests, which is quickly followed by increased population density, logging, and minor road development [20]. As such, distance to population center can be used as a proxy for LUC; regions void of significant road development are often characterized by more intact forest [21]. Small forests, fragments and patches, are well-studied and known to have strong abiotic and biotic responses to decreasing forest size. Edge effects increase temperature, light, wind, and disturbance, while decreasing humidity. These effects permeate up to 300 m into the forest [22] and have been associated with negative effects on taxon abundance, diversity, carbon storage, and ecosystem services in general [23].

While landscapes characterized by large, intact forests are best suited to sustain high levels of biodiversity and provide a constant supply of multiple ecosystem services [15, 24, 25], other landscapes have been so heavily transformed that a mosaic of small forest patches is all that remains to serve as an ecosystem refuge. These areas cannot immediately transition back to large intact, mature forests and, thus, the overall health of the forest mosaic is critical to avoid further declines in biodiversity. This is seen acutely in northern Ethiopia where the last remaining forests are small patches that average 5 ha in size [26, 27]. However, while small in size, in South Gondar alone (14,607 km^2^) there are 1022 of these forests that span elevations and biomes making up ~ 5500 ha of tree cover (Scull, unpublished data). These forests have a church at their center and are considered critical in protecting the sacredness of the church and as such their association with the church protects them [28]. This protection due to sacredness is often referred to as shadow conservation [27, 29]. These church forests in northern Ethiopia have shown remarkable persistence; 99% of forests have survived the past half-century, with no forest found without a church [27]. However, the density of scattered trees and bushlands that surround these forests has declined over the past 80 y, which amplifies forest isolation and edge effects [4]. As a result of declining woody biomass outside of the forests, the demand on the forest for both material and non-material use has likely increased, which could compromise the integrity and persistence of these forests on the landscape. Previous work in the region has found that over a 50 y time span, crown closure declined significantly and 37% of the total forest area was characterized by some form of disturbance [27].

This study principally aimed to determine the impacts of human disturbance (e.g. human structures, planted taxa, weedy taxa) on church forest integrity and regeneration potential. In addition, we examined the factors of forest size, elevation, distance to population center, and the presence of an exterior wall on species richness, density (ha^-1^), and biomass (ha^-1^) of standing live trees, as well as the richness and density of tree seedlings in 44 church forests across South Gondar, Ethiopia. Previous research indicates that the presence of a wall, established for various reasons including demarcating land and blocking human and animal access, can have a protective effect on forests as their presence increased seedling richness and density, but did not affect tree species richness or density [6]. As such, we include the presence of walls in our analysis of seedling richness. We hypothesized that human disturbance would negatively affect tree species richness, density, biomass and seedling regeneration and have a greater impact in smaller forests that were closer to a population center. This research is part of a long-term study on the factors that contribute to the effective management of church forests in Ethiopia.

## Materials and methods

### Study area and site selection

**Fig 1:**
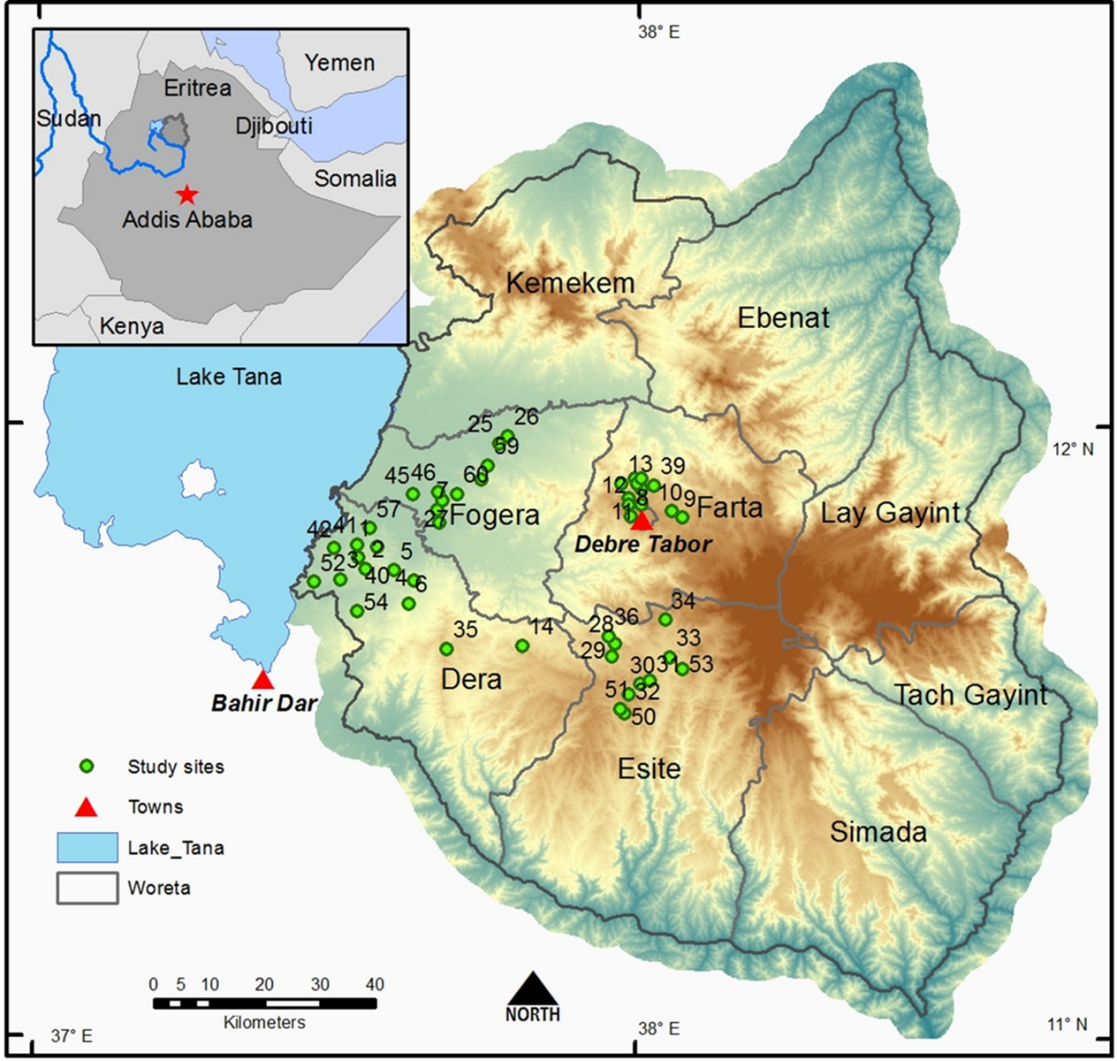
Map of study area in the South Gondar region of northern Ethiopia. Description: Church forests are scattered in the landscape and are separated by ~ 2 km with a matrix of agricultural land separating them. Named areas are Woredas or Districts [50]. Basemap images obtained form ESRI.

**Fig 2:**
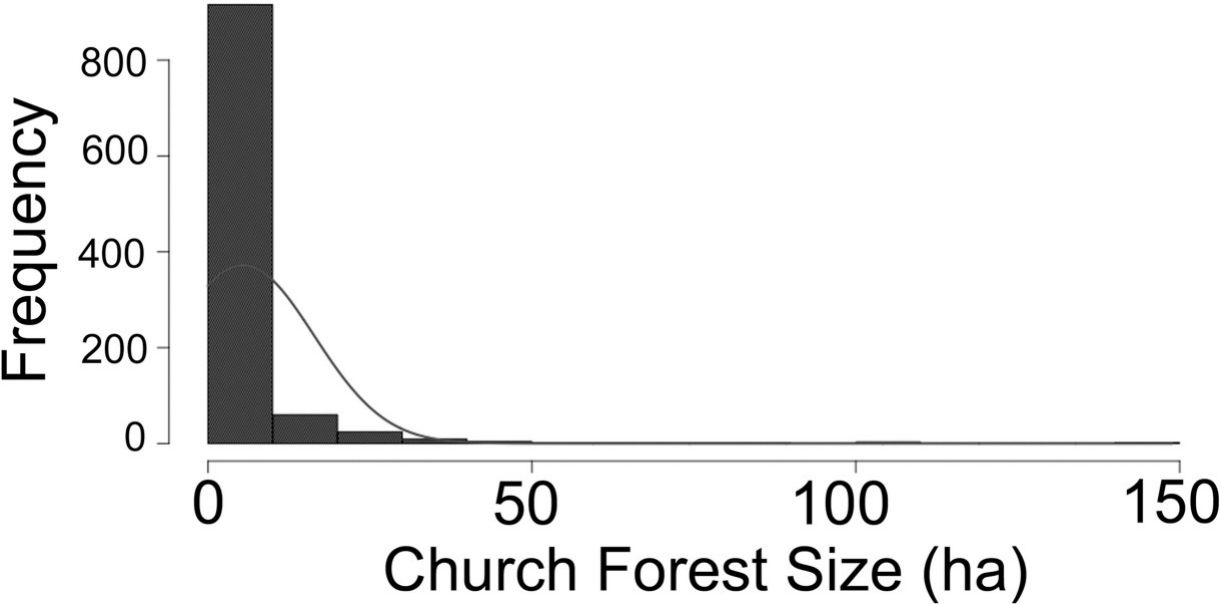
Average size of church forests in the region is small, 5.42 (+/- 0.34 ha). There are 1022 church forests in South Gondar, Ethiopia, an area of 14,059 km^2^. Forest range from <1–148.7 ha, 93% of these forests are < 15 ha [27].

We established long-term research sites in 44 church forests in the tropical, seasonally dry montane region of South Gondar in northern Ethiopia. The average annual rainfall is 700–800 mm with most falling June–August (Nyssen et. al. 2005). The distribution and abundance of the church forests in the region are detailed in another paper [27]. In brief, there are ~1022 church forests in South Gondar, with no forest detected without a church. The forests are scattered throughout the landscape separated by approximately 2.10 km (± 0.03) ha and are small on average, 5.42 (± 0.34; range 0.02–148.86) ha [27] (Figs 1 & 2). To access church forests, we secured permission from the office of Abune Aregawi, Bishop of South Gondar, which gave us permission letters for entry to each church forest.

**Fig 3:**
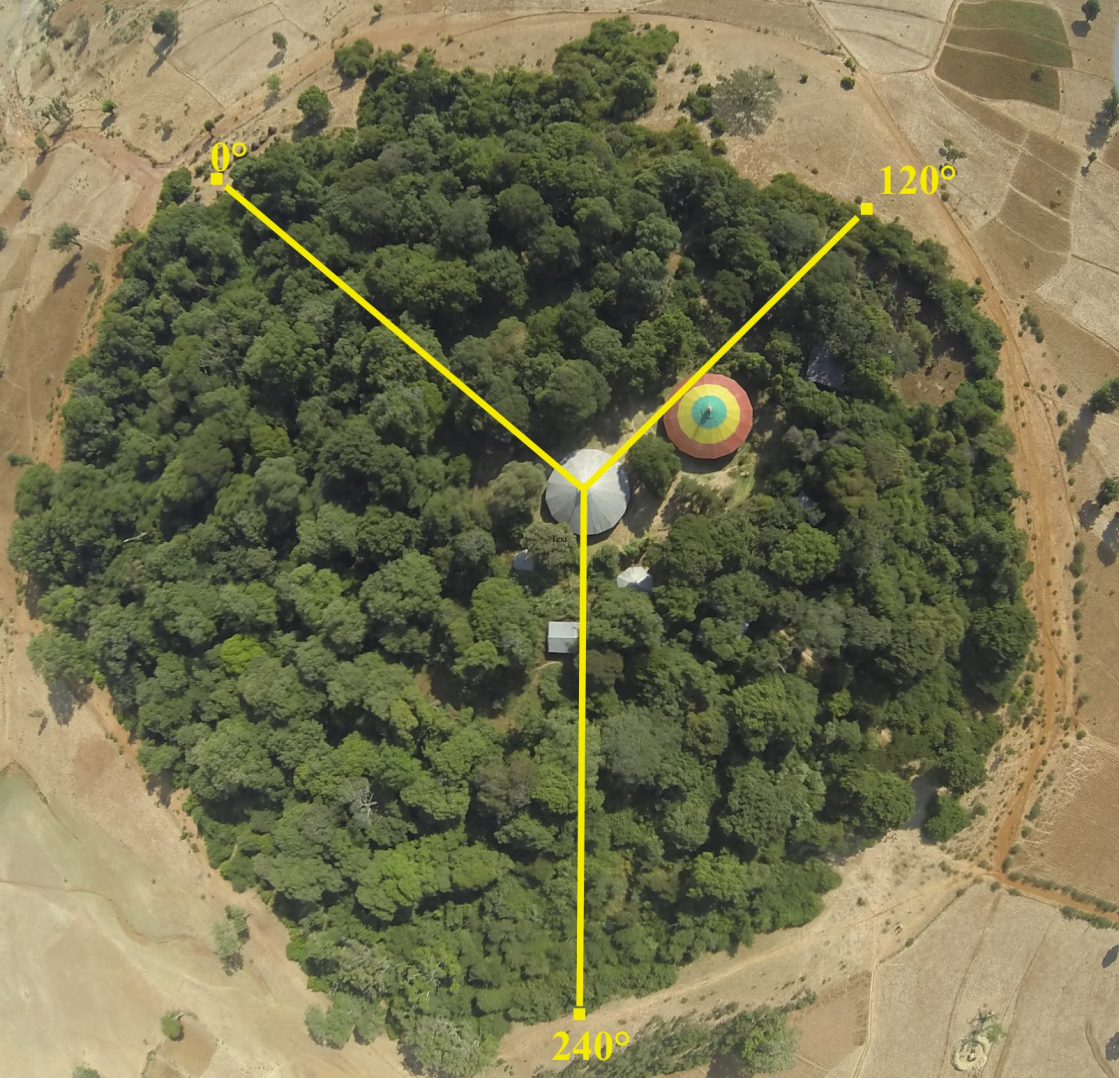
Transect design for the quantification of plant species richness, density, and biomass in 44 church forests in northern Ethiopia. Modified Gentry transects were established in each forest at three cardinal directions (0°, 120°, and 240°) from the church center out towards the edge of the forest. Each transect was 2 m wide and the length depended on the size of the forest. Within each transect every stem > 1 cm was measured and identified as well as any type of disturbance recorded.

These forests are sacred as they surround a Christian Orthodox Tewahido Church [26, 28, 30]. The church itself is usually in the center of the forest within a walled clearing (Fig 3). These church forests are active with religious services and events throughout the week [28]. Along the edges of the forests are mahabirs, or gathering areas, which are maintained open spaces that are used for meetings and other events [28]. These mahabirs along with trails, graves, and small huts contribute to the constant human presence within forests, which could influence the integrity of the forest. In addition, often along the edges there are plantings of both native and exotic taxa [27]. Sometimes these forests have a wall surrounding the perimeter of the forest that is made with local rocks; these walls all vary in height, age, and intactness.

The church forests of Ethiopia span elevations and biomes, with montane region (1800–2050 m a.s.l.) characterized by evergreen broadleaf taxa *Prunus* (Rubiaceae) and *Mimusops* (Sapotaceae) and the upper montane region (2400–2700 m a.s.l) characterized by *Juniperus* (Cupressaceae) and *Euphorbia* (Euphorbiaceae) [27]. To determine the effects of disturbance, forest size, distance to population center, elevation, and the presence of a wall on forest status, we selected 44 forests that ranged in size between 1.4–15.4 ha, both above and below the mean size, but still representative (Table 1). Distance was categorized as either near (< 50 km) or far (> 50 km) from two cities, Bahir Dar (population 243,330) for montane church forests or Debre Tabor (population 87,100) for upper montane church forests [31]. Sample church forest number is not uniform for all categories, however; we have at least four forests within each category. Forest properties assessed were: human disturbance, rarefied species richness, tree density, and biomass ha^-1^; and seedling richness and density.

**Table 1:** The 44 study forests in relation to elevation, distance to population center, and the presence of a wall.

Elevation	Montane (1800–2050 m)		Upper Montane (2400–2700 m)	
Distance from Bahir Dar (montane) or Debre Tabor (upper montane)	Wall	No Wall	Wall	No Wall
Near (< 50 km)	4	7	6	5
Far (< 50 km)	5	6	7	4

Sample number of forests within each study category. Forest sizes ranged from 1.4–15.4 and were represented across all categories.

### Transect Methods: Disturbance, species richness & density, biomass

Modified Gentry transects [27, 32] were established in each forest at three cardinal directions (0°, 120°, and 240°) from the church center to the edge of the forest (Fig 3). Each transect was 2 m wide and the length depended on the size of the forest. Along each transect, all woody species greater than 1 cm were identified, measured for diameter at breast height (dbh), and recorded. Biomass of trees was calculated using the same allometric relationship because species-specific functions were not available (Eq 1).

34.4703−8.0671*(dbh)+0.6589*(dbh*dbh). (Eq 1)

Native taxa were noted along each transect in addition to the type and location of any form of disturbance across four categories (Table 2). While our aim was to note disturbance, both natural (e.g. gaps, tree falls) and human (e.g. clearings), we quickly discovered that there was little to no natural disturbance and we were documenting human disturbance. The percent of native taxa and each disturbance type along the transect was calculated (Eq 2).

$$\frac{area\;of\;human\;disturbance\;type}{area\;of\;transect}*100$$ (2)

**Table 2:** Type of human disturbance along study transects.

Type of Human Disturbance	Description
Weedy Taxa	*Acanthus* and *Justica* are two common and dense weeds in the forest that concentrate along the edges and within gaps.
Planted Taxa	Exotic: *Eucalyptus*: Plantations of *Eucalyptus* are found both along edges and within some forests. This is used for building material and for profit.
	Native taxa include: *Juniperus*, used for buildings within the forest; *Coffea*, coffee which is often sold; and *Rhamnus*, used for making the traditional dark beer, Tella.
Non-planted Exotic Taxa	Although rare, these are exotic species found dissociated from plantations
Other Human Disturbance	Buildings, graves, slashed trees, evidence of grazer forage, gathering areas, clearings.

Human disturbance categories that were quantified across transects in 44 church forests in northern Ethiopia. Disturbance types are categorized as natural or human.

Along each transect at three points (in the center, 10 m from the outer edge, and 10 m from the internal wall around the church), we measured seedling richness and density in 1 m^2^ quadrats for a total of 3 seedling plots per transect and 9 seedling plots per site.

### Statistical analysis

We used Model 1 Fixed Effects Analysis of Variance (ANOVA) to determine if size, distance to population center, elevation, and the presence of a wall were significantly associated with each of our dependent variables, namely: tree species richness, trees species density, tree biomass, seedling density, seedling richness, and disturbance [34]. We used regressions to determine if there were causative linear relationships between our independent and dependent variables. Because disturbance was pervasive across sites, we also examined the association of disturbance with the dependent variables in ANOVA and Regression models. Normality of model residuals for both regression and ANOVA were tested using the Shapiro-Wilk Normality Test, and all models presented conform to normality assumptions for ANOVA and Regression analysis (*p* > 0.05).

To compare tree species richness among church forests, we used rarefied species richness to control for the variation in the number of individual trees found among our sites. We used the rarefy function in the vegan package (v. 2.5–3, [35]) in R (v. 2.51, [33]) to generate rarefied tree species richness.

## Results

Across 44 church forests, we identified and measured 11,310 trees > 1 cm and identified 139 species (excluding unknowns) in 105 genera and 69 families and found an average species richness, excluding exotic and planted taxa, of 10.8 (± 0.5). The most species rich plant families were: Fabaceae, Asteraceae, Rubiaceae, Celastraceae, Euphorbiaceae, Lamiaceae and Malvaceae.

### Disturbance

**Fig 4:**
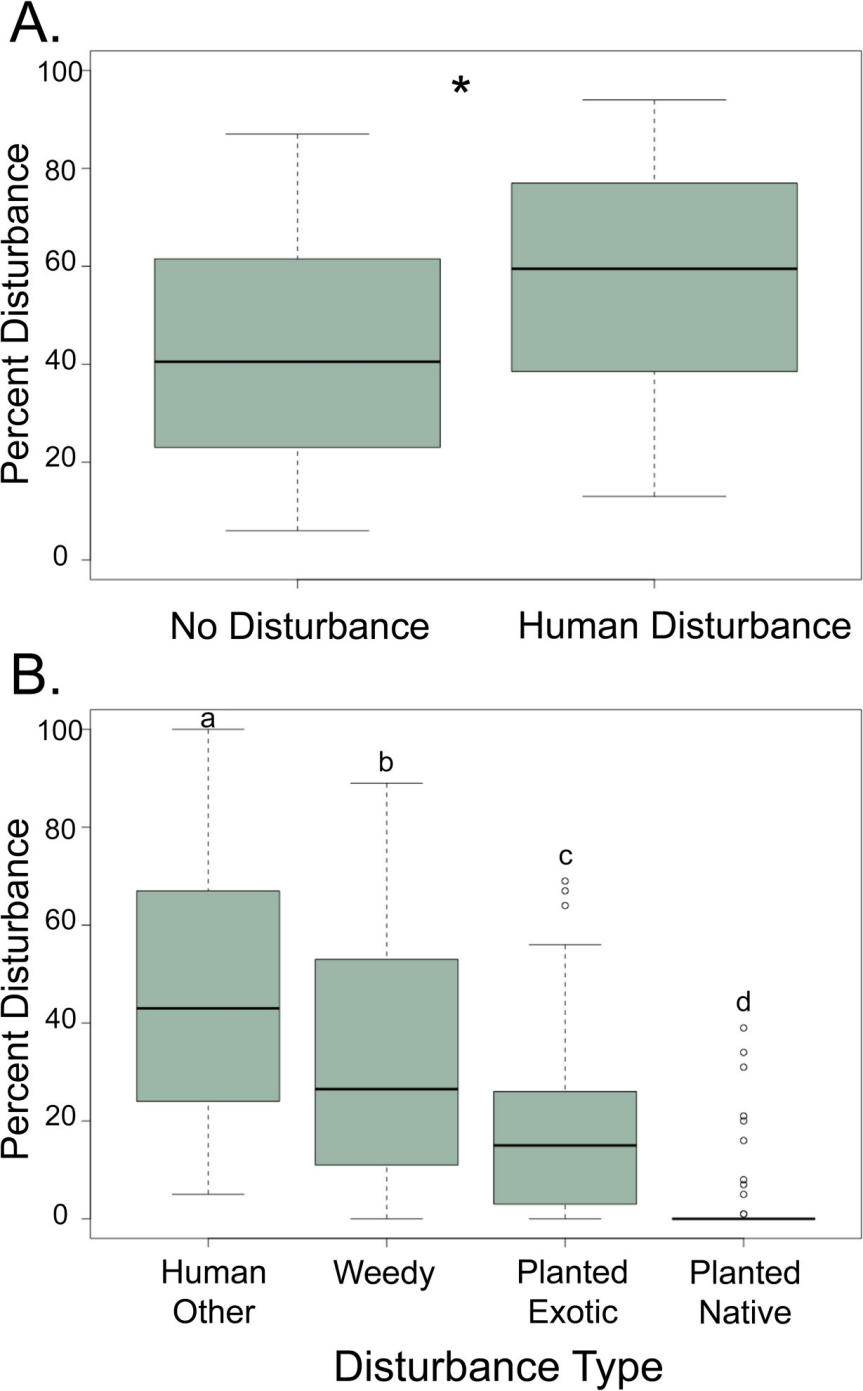
Human disturbance is pervasive across church forests. **A.** Box-and-whiskers plots of percent no disturbance and human disturbance across 44 church forests in northern Ethiopia. Asterisk indicates significant difference using a student’s t-test (*t* = 3.18, *p* = 0.002). **B. Human disturbance types across church forests.** Box-and-whisker plots of disturbance types in church forests. Lower case letters indicate significant differences among categories using a Tukey HSD, *p* < 0.05. The majority of disturbance in church forests is from direct human impacts followed by the proliferation of weedy species.

Human disturbance was pervasive across sites, with an average total disturbance of 56.2% (± 3.2; Fig 4). When broken down by disturbance types, human-induced disturbance due to buildings and clearings was significantly higher than human disturbance due to the introduction of weeds and the planting of exotic or native species (ANOVA: *F*_3,180_ = 33.02, *p* < 0.001; Fig 4). We found that disturbance was not significantly associated with forest size, distance to population center, elevation, or the presence of a wall (ANOVA: *F*_4,39_ = 1.888, *p* = 1.34). However, disturbance had a weak but significant, negative linear relationship with forest area (*F*_1,42_ = 4.32, R^2^ = 0.10, *p* = 0.047).

#### Species richness

**Fig 5:**
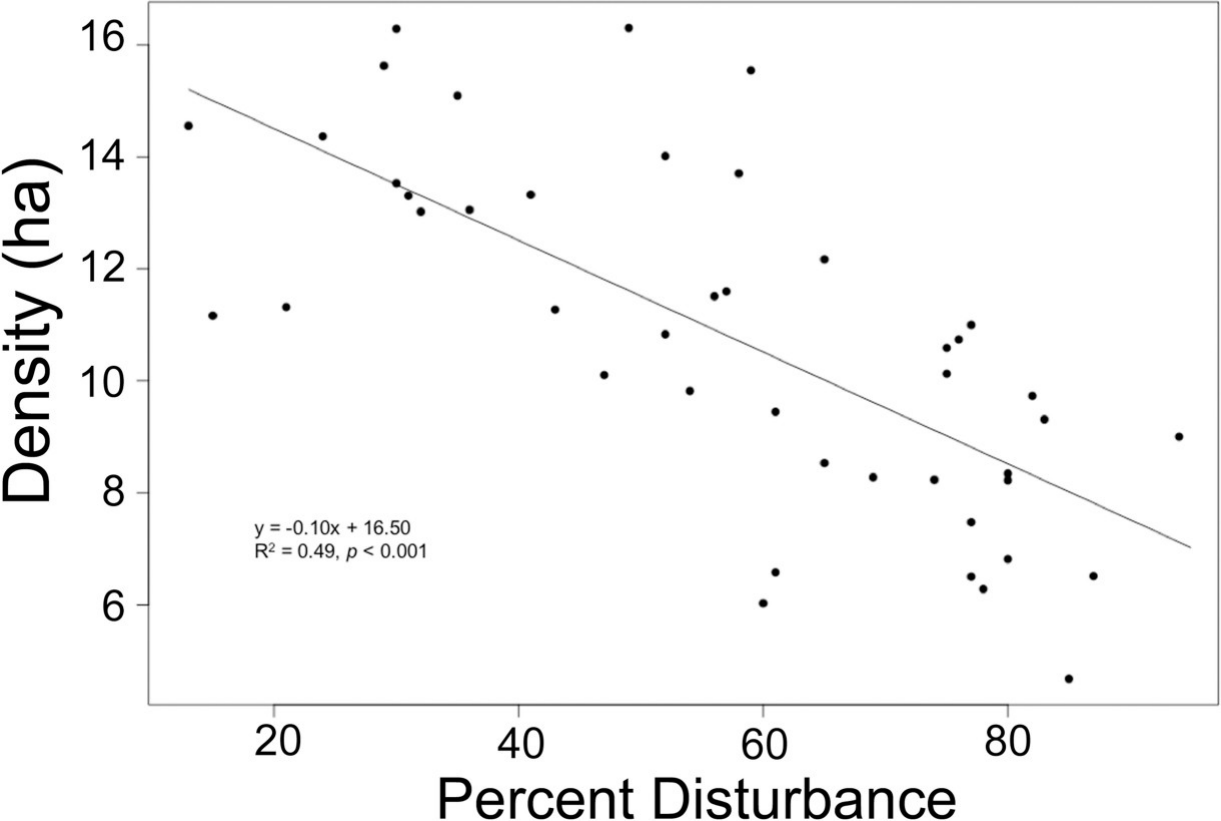
Species richness decreases significantly with increasing percent disturbance. The relationship between tree species richness and percent forest disturbance in 44 church forests in northern Ethiopia.

We found that species richness was significantly associated with forest size and disturbance, but not elevation, distance to population center or the presence of a wall (Table 3). Species richness had a significant, negative relationship with disturbance across all sites (Fig 5).

**Table 3:** ANOVA results on the effect of church forest disturbance, size, distance to population center, the presence of a wall, and elevation on species richness (ha^-1^), density (ha^-1^), and biomass (ha^-1^).

	Species Richness (ha^-1^)			Density (ha^-1^)			Biomass (ha^-1^)		
	df	*F*	*p*	df	*F*	*p*	df	*F*	*p*
Model	5,38	14.98	**< 0.001**	5,38	5.50	**< 0.001**	5,38	5.66	**< 0.001**
Disturbance	1	35.00	**< 0.001**	1	11.61	**0.002**	1	26.47	**< 0.001**
Size	1	37.66	**< 0.001**	1	7.49	**0.009**	1	0.003	0.956
Distance	1	1.28	0.262	1	0.06	0.802	1	0.324	0.573
Wall	1	0.86	0.361	1	1.01	0.350	1	0.744	0.394
Elevation	1	0.07	0.795	1	7.30	0.010	1	0.745	0.395

Church forest disturbance and size were the factors that were most strongly associated with species richness (ha^-1^), density (ha^-1^), and biomass (ha^-1^).

#### Density

**Fig 6:**
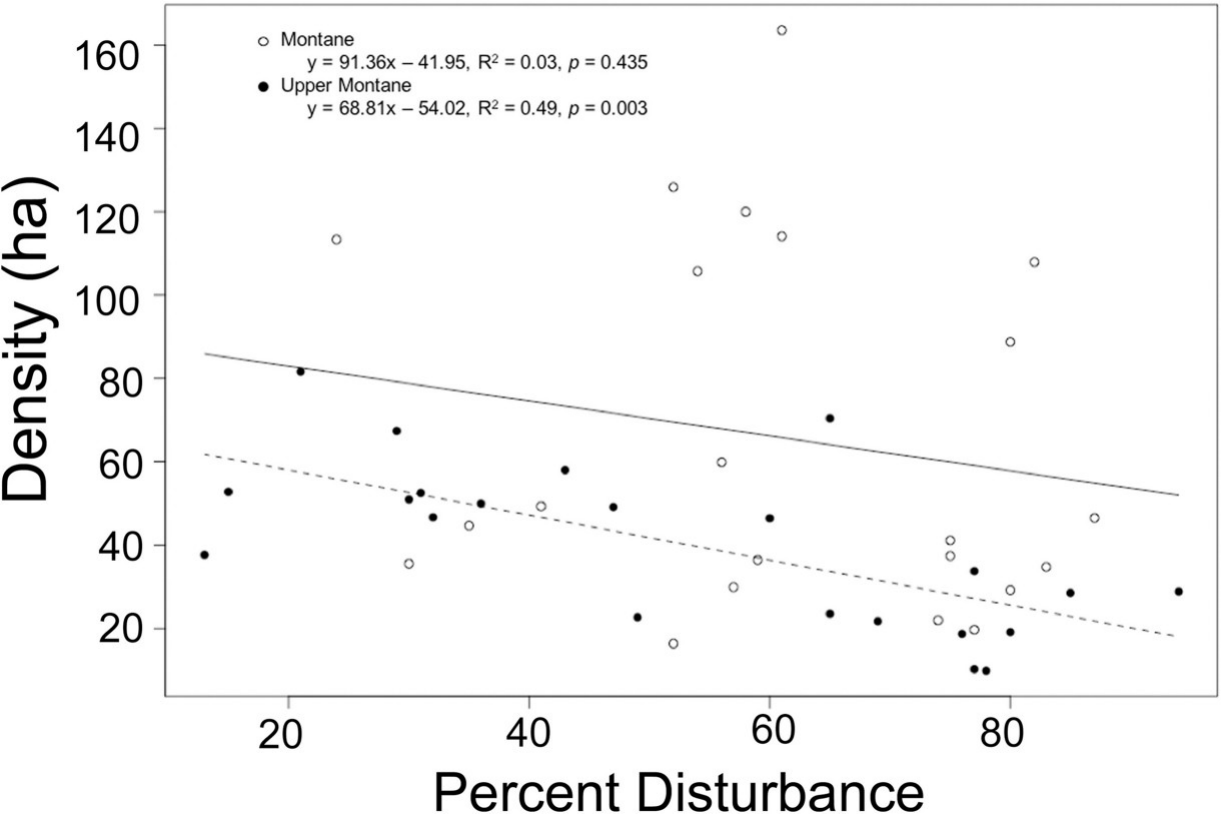
Tree density decreases with increasing percent disturbance in upper montane forests. Description: Tree density (ha^-1^) decreased significantly with increasing percent disturbance in the upper montane forest sites, however; density did not vary significantly with disturbance in the montane sites.

Tree density (ha^-1^) was significantly associated with forest size, disturbance, and elevation, but was not associated with distance to population center or the presence of a wall (Table 3). Tree density (ha^-1^) was significantly higher in the montane region (65.5 ± 9.2) than the upper montane region (40.03 ± 4.45; *t* = 2.52, *p* = 0.017). While disturbance did not vary significantly between elevations (*t* = 1.26, *p* = 0.214), tree density in montane forests did not vary with disturbance whereas upper montane forest tree density varied significantly with disturbance (Fig 6).

#### Biomass

Disturbance was the only factor that significantly explained biomass (ha^-1^) in the full ANOVA model (Table 3). As with other variables, there was a significant, negative relationship of biomass with disturbance (*F*_1,42_ = 19.72, R^2^ = 0.30, *p* < 0.001).

#### Seedlings

Seedling density (2.1 ± 0.33) and richness (0.66 ± 0.09) were low across all sites. We found that there was a significant effect of a wall, forest size, and a significant wall * size interaction on seedling richness (Model: *F*_3,40_ = 5.35, *p* = 0.034; wall *F*_1_ = 7.15, *p* = 0.018; forest size: *F*_1_ = 5.01, *p* = 0.031; wall * size, *F*_1_ = 5.66, *p* = 0.022) and a marginally significant effect of wall and wall * size interation, but no effect of forest size, on seedling density (Model: *F*_3,40_ = 3.06, *p* = 0.039; wall *F*_1_ = 3.82, *p* = 0.058; forest size: *F*_1_ = 2.27, *p* = 0.139; wall * size, *F*_1_ = 3.99, *p* = 0.052). In church forests with a wall, we found a significant positive relationship between seedling richness and density with forest size (seedling richness: *F*_1,20_ = 9.98, R^2^ = 0.30, *p* = 0.005; seedling density: *F*_1,20_ = 5.35, R^2^ = 0.17, *p* = 0.032), but no significant effect of either without a wall (*F*_1,20_ = 0.01, R^2^ = 0.05, *p* = 0.920; *F*_1,20_ = 0.12, R^2^ = 0.04, *p* = 0.714).

## Discussion

Our research on 44 sacred Ethiopian church forests across elevations and distances from population center indicate that more than half of the forest area showed human disturbance, which was the key factor, followed by size, influencing species richness, biomass (ha^-1^) and density (ha^-1^) of species. The forest clearings are important for human gatherings and other activities [28], however; this use can further increase disturbance and decreases the opportunity for seedling establishment and regrowth. The dominance of weedy species is also problematic for forest regeneration as weedy species, such as *Justica* and *Acanthus* (Acanthaceae), have been shown to outcompete tree species hindering regeneration [36, 37].

When a disturbance occurs in a small forest every disturbance becomes a higher percentage of the whole. Small forests are more susceptible to edge effects and tree mortality than larger forests and these effects can have a large, negative impact on their resilience and long-term persistence [38, 39]. With increasing disturbance, species richness, biomass (ha^-1^) and tree density (ha^-1^) decreased significantly and this was exacerbated by decreasing forest size. In a survey of 78 church forests in the northern highlands of Ethiopia, the average size was even smaller than found in our study (~2 ha, [40, 41]), and they found that their forests had > 50% of the tree species richness found in tropical northeastern Africa. In tropical lowland forests in central Amazonia, rates of mortality of large canopy trees (dbh > 60 cm) were significantly higher in edges (up to 300 m) than in interior forest (> 300 m into the forest) (Laurance et al. 2000). Thus, in small church forests where edge effects likely influence the entire forest, large canopy trees, which are often the seed sources for forest regeneration, are compromised.

Tree density (ha^-1^) was the only variable influenced by elevation, with upper montane forest density decreasing with increasing disturbance while montane forest density did not vary significantly (Fig 5). The montane church forests had more variable disturbance than the upper montane forest which may reflect differential use by local people. These differences may also reflect the different plant life zones within each elevation, as the montane forests are represented by more dense evergreen broadleaf plant taxa such as *Teclea* (Rutaceae) and *Mimusops* (Sapotaceae), whereas the upper montane region has more sparcely dispersed taxa such as *Juniperus* (Cupressaceae) and *Euphorbia* (Euphorbiaceae).

Consistent with previous research, the presence of a wall had a significant effect on seedling number and richness across sites [6], however; the differences in these numbers was small. These findings suggest that while a wall may have a positive influence on seedling communities, the regeneration potential of these forests is compromised by the magnified effects of disturbances on the adult trees in these small forests. Lower species richness could reduce the ability of these forests to recover from disturbance and lower density could compromise the cultural services provided by the forest to the community, such as shade and cover for the church [28].

Our data do not support research indicating that increasing isolation, as measured by distance from population centers, is associated with decreased human disturbance [42]. In fact, our data indicate that forests < 15.5 ha show no difference in disturbance with distance. One reason for the lack of difference between forests close to population centers vs. those > 50 km from a population center may be related to the high rural population in Ethiopia, 80% [43], that may be reliant on forests for their basic needs (e.g. firewood, honey, etc.) compared to their city-dwelling counterparts. Thus, demand may be similar in both areas, in the city because of high population numbers and in the rural areas because of need. It could also be that urban timber needs are being met by *Eucalyptus*, which is more readily available in church forests near population centers [27].

While these church forests have exhibited remarkable persistence over the last 50–80 years, on average only 0.42 ha smaller 50 years ago [4, 27], recent work on the matrix between forests shows that the buffer, shrubs and trees, around forests has decreased significantly [4]. The loss of a buffer increases edge effects, such as lower relative humidity, higher temperatures and higher winds [23] and increases mortality rates of seedlings [9, 44]. These church forests are at risk of being Janzen’s [1] ecologically “living dead” (*i*.*e*., forests that last only until the large trees die as they do not have any regeneration) due to their increasing isolation combined with high disturbance and low seedling recruitment [6, 45, 46].

While seemingly stagnant in recruitment of seedlings and high disturbance, these forests are alive with human activity as they are gathering areas for meetings, worship, and schooling for the local people [27, 28, 47]. Conservation initiatives usually prioritize biodiversity and ecosystem function and not human use, however; if this approach is taken in South Gondar, few forests would be protected as there are very few large forests, most notably there are only 9 forests > 50 ha (Fig 2), which is why we focused our study on forests that were the average size. In fact, in the 14,059 km^2^ area of our study, there are 1022 forests with 93% of them < 15 ha (Fig 2; [27]). Unfortunately, continued deforestation around the world is increasing forest fragmentation, increasing their proximity to edges [11], and highlighting the need for conservation efforts directed toward forest fragments.

These church forests, even when small, are important refugia for trees and biodiversity in general [41, 48], however; their continued persistence on the landscape is threatened without active conservation efforts [11]. We recommend that conservation priorities not only protect large forests, but also the small and highly used forests which are critical areas for local people. Previous research and local initiatives have found that building a wall increases seedling species richness and density, likely because it not only reduces access to the forest by grazing cattle, but also because it directs people to use the trails rather than randomly entering and walking through the forest [6]. However, as our findings and those of others show [40, 41], seedling recruitment and diversity with walls is still not high and most species are not represented [49]. Thus, walls alone are not enough and solutions that protect and enhance forest regeneration potential while also preserving cultural needs, such as active planting programs both inside and between the forests, the removal of exotic and weedy species, discouraging the creation of new paths and clearings, and maintaining large trees as they are important seed sources, must be implemented. The interdependence between the forest and the church community make conservation efforts essential.

## Supporting information

S1 Appendix Data on 44 sacred church forest study sites. Data on sacred church forests include: Woreda, Kebele, size, distance to population center, elevation, the presence of a wall, tree species richness, trees species density, tree biomass, seedling density, seedling richness, and % of disturbance types.
(XLSX)
